# Vaccines to Prevent Infectious Diseases in the Older Population: Immunological Challenges and Future Perspectives

**DOI:** 10.3389/fimmu.2020.00717

**Published:** 2020-04-23

**Authors:** Angelika Wagner, Birgit Weinberger

**Affiliations:** ^1^Department of Pathophysiology, Infectiology, and Immunology, Institute of Specific Prophylaxis and Tropical Medicine, Medical University of Vienna, Vienna, Austria; ^2^Institute for Biomedical Aging Research, Universität Innsbruck, Innsbruck, Austria

**Keywords:** immunosenescence, elderly, vaccine, high-dose, adjuvant, influenza, *Streptococcus pneumoniae*, herpes zoster

## Abstract

Infectious diseases are a major cause for morbidity and mortality in the older population. Demographic changes will lead to increasing numbers of older persons over the next decades. Prevention of infections becomes increasingly important to ensure healthy aging for the individual, and to alleviate the socio-economic burden for societies. Undoubtedly, vaccines are the most efficient health care measure to prevent infections. Age-associated changes of the immune system are responsible for decreased immunogenicity and clinical efficacy of most currently used vaccines in older age. Efficacy of standard influenza vaccines is only 30–50% in the older population. Several approaches, such as higher antigen dose, use of MF59 as adjuvant and intradermal administration have been implemented in order to specifically target the aged immune system. The use of a 23-valent polysaccharide vaccine against *Streptococcus pneumoniae* has been amended by a 13-valent conjugated pneumococcal vaccine originally developed for young children several years ago to overcome at least some of the limitations of the T cell-independent polysaccharide antigens, but still is only approximately 50% protective against pneumonia. A live-attenuated vaccine against herpes zoster, which has been available for several years, demonstrated efficacy of 51% against herpes zoster and 67% against post-herpetic neuralgia. Protection was lower in the very old and decreased several years after vaccination. Recently, a recombinant vaccine containing the viral glycoprotein gE and the novel adjuvant AS01B has been licensed. Phase III studies demonstrated efficacy against herpes zoster of approx. 90% even in the oldest age groups after administration of two doses and many countries now recommend the preferential use of this vaccine. There are still many infectious diseases causing substantial morbidity in the older population, for which no vaccines are available so far. Extensive research is ongoing to develop vaccines against novel targets with several vaccine candidates already being clinically tested, which have the potential to substantially reduce health care costs and to save many lives. In addition to the development of novel and improved vaccines, which specifically target the aged immune system, it is also important to improve uptake of the existing vaccines in order to protect the vulnerable, older population.

## Changes of the Immunological Network in Aged Individuals

Demographic changes lead to global aging of the population and the percentage of persons older than 65 years is projected to increase from 9% in 2019 to 16% in 2050 worldwide and from 18 to 25% in Europe and Northern America. The number of people above age 80 is growing even faster (1). The severity of many infections is higher in older compared to younger adults and infectious diseases are frequently associated with long-term sequelae such as onset of frailty, impairments in activities of daily living, or the loss of independence (2, 3). The prevention of infectious disease is an important measure to ensure healthy aging and improve the quality of life, and vaccination is the most promising strategy to achieve this goal. However, most currently available vaccines are less immunogenic and effective in older compared to young adults. With age, the immune system undergoes characteristic changes, which lead to functional deficits and dysregulation of most immune mechanisms. Alterations in the function of innate immune cells at the site of injection are particularly relevant for vaccine induced immune responses. Neutrophils contribute to a pro-inflammatory environment at the site of vaccine injection, thereby recruiting and activating other innate immune cells, e.g., monocytes/macrophages and dendritic cells (DC). Reduced chemotaxis, alterations in signal transduction following antigen recognition and aberrant cytokine production have been described for neutrophils derived from older persons (4). Similar deficits have been observed for monocytes/macrophages and DCs, which are also impaired in their capacity to process and present antigen to T cells e.g., due to deficits in the upregulation of MHC-proteins and costimulatory molecules (5). Adjuvants are used to stimulate innate immune responses and are a promising strategy to overcome age-associated limitations, as detailed below. The composition of the T cell compartment changes substantially with age due to alterations in hematopoiesis and to thymic involution. With dramatically decreased output of newly generated naïve T cells, the portion of these cells shrinks with age, limiting responses to neo-antigens. In contrast, antigen-experienced, particularly repeatedly stimulated, highly differentiated T cells accumulate. Their diversity is restricted, they produce preferentially pro-inflammatory cytokines and show a diminished response to antigenic stimulation (6–8). For many vaccines, T cell responses are relevant for protection and deficits directly impact vaccine efficacy. In addition, T cell help provided by follicular helper T cells in the germinal center is crucial for optimal antibody responses. Specific age-related deficits of this cell type have recently been summarized elsewhere (9). Similar to the T cell compartment the composition of the B cell pool also changes with age and more autoreactive and less affine B cells can be observed. Intrinsic defects of B cells, such as reduced somatic hypermutation and isotype switch as well as reduced numbers of plasma cells contribute to reduced antibody responses after vaccination (10). An extensive review of immunosenescence is beyond the scope of this article, but can be found elsewhere (11, 12).

There are many factors in addition to chronological age, which influence immune responses to vaccination. Underlying co-morbidities, obesity, and frailty have been shown to be associated with lower immune responses to various vaccines in many studies. A comprehensive review of these aspects is beyond the scope of this manuscript, but can be found elsewhere (13, 14). Several age-related chronic diseases, such as cardiovascular disease and chronic obstructive pulmonary disease (COPD) are risk factors for infections, but at the same time are also associated with lower immune responses to vaccination. As an example, patients with congestive heart failure or COPD develop lower antibody concentrations against influenza vaccine (15, 16). *Post hoc* analysis of the CAPiTA study, which investigated the efficacy of the 13-valent conjugated pneumococcal vaccine, showed that 80% of the pneumonia cases, which occurred in the unvaccinated placebo arm of the study population, affected persons with one or more co-morbidities (e.g., asthma, diabetes, chronic heart, liver, or kidney disease). The incidence of community-acquired pneumonia in this at-risk population was 4.2 times higher compared to healthy individuals. Efficacy of the vaccine against first episodes of vaccine-type pneumonia was reduced to 40.3% in the at risk cohort compared to 66.7% in the healthy participants (17).

Cytomegalovirus is a highly prevalent β-herpesvirus, which establishes lifelong latency after primary infection. Latent CMV-infection has a profound impact on the composition of the T cell compartment (18) as well as on other immune cells, such as NK cells (19). Some studies showed a link between CMV-infection and reduced survival in very old age (20) as well as with cardiovascular disease and other inflammation-related diseases (21, 22). Antibody levels or CMV-seropositivity were associated with limited responses to influenza vaccination in some studies (23, 24) and long-term persistence of diphtheria-specific antibodies was lower in CMV-positive older persons compared to CMV-negative individuals (25). However, several studies also demonstrated the absence of a CMV-related effect on vaccine-induced immune responses (26).

In this review we will discuss the benefits and limitations of currently available vaccines designed for the older population (influenza, pneumococcus, herpes zoster) and key strategies, which have been tested or are under development in order to enhance vaccine responses in the older population.

## Influenza: How to Protect Against a Changing Virus

Influenza virus is transmitted via direct contact, droplets and fomites. This virus targets respiratory epithelia leading to lung inflammation and resulting in an acute respiratory infection. After a short incubation time of on average 1 to 2 days symptoms occur with fever and cough being most prominent. The course of disease may range from asymptomatic and mild self-limiting disease to severe course, where patients require hospital care. Particularly the burden of severe disease and mortality is increasing with age and is highest in those above 75 years (27). Due to immunosenescence, susceptibility, disease severity as well as complications such as bacterial co-infections and exacerbation of chronic pulmonary diseases are raised leading to higher frailty and mortality with age (28). Therefore effective protection by vaccination is desirable. Unfortunately, efficacy of the currently used vaccines reaches at best 50% in this risk group (29, 30). Key issues are that vaccine efficacy is imperfect even at younger age reaching at maximum 70% in preventing laboratory-confirmed influenza in placebo controlled studies (31, 32) and depends not only on age, but also on season, strain, vaccination, and infection history (33).

In the older population, either split or subunit influenza vaccines are currently used for prevention. Split virus vaccines contain disrupted viral envelopes that lost infectivity but retained immunogenicity. Subunit virus vaccines are produced by further purification steps that remove the nucleocapsid from the split virus (34). One of the reasons for developing split and subunit vaccines was to decrease reactogenicity (35), however, in light of different adjuvants that may be added this effect is not always evident (36). Virosomal vaccines, consisting only of the virus envelope, have not been available for the past influenza seasons and live attenuated vaccines are not licensed for the elderly due to safety concerns.

Human influenza disease can be caused by influenza A and B strains. Previously influenza vaccines were usually trivalent, consisting of two circulating influenza A strains (one H1N1 and one H3N2 strain) and one influenza B strain either of the Victoria or Yamagata linage. Since both B linages have been shown to co-circulate, tetravalent vaccines including both B linages became increasingly available in the last years to optimize coverage (37).

With age influenza-specific antibody titers decline faster leading to loss of seroprotection until the following season and possibly rendering vaccinees susceptible to some influenza strains even toward the end of the same season (38, 39). To overcome the lower influenza efficacy in the elderly high-dose, adjuvanted and intradermally administered vaccines have been developed and are in clinical use. Depending on the availability these enhanced vaccines are widely recommended in national guidelines with the adjuvanted vaccine being the preferred vaccine for those over 65 years in countries such as Austria and the United Kingdom (40, 41).

It is also the first exposure to influenza vaccines and/or infection that – according to the theory of the original antigenic sin – imprints immune responses for the following antigen encounters with drifted variants irrespective of age. Thus, contact with drifted virus strains may rather boost immune responses to epitopes shared with the previously encountered strain(s) than inducing antibody responses toward the new antigenic determinants thereby resulting in a lower vaccine efficacy (42). Related to this theory there is data showing that vaccine efficacy was reduced in individuals that had been vaccinated also in preceding seasons compared to those vaccinated only in the current season, though this effect was neither seen consistently for all strains nor for different seasons in different study populations (43). Low vaccine efficacy independent of age can also result from antigenic mismatch of circulating versus vaccine strains due to antigenic drift that may occur during one season (44). Therefore vaccine composition is adapted every year and annual revaccination is required.

Additionally, IgA, which would be able to inhibit viral cell entry and therefore infection, is only poorly induced by intramuscularly (i.m.) administered vaccines, and this further contributes to limited clinical efficacy (45).

It is important to note, that hemagglutinin antibodies are widely used as surrogate of protection for influenza vaccines and also for the licensure of vaccines (46). However, HA antibodies might not be an ideal measure in older adults as vaccinees with low titers may be still protected and vice versa (47). Along these lines, memory B cells and plasmablasts are retained in the elderly, while antibody titers are lower compared to young even after repeated vaccination, which has been proposed to be a consequence of impaired differentiation from memory B cells toward plasma cells (48). Additionally, cell-mediated immune responses are important to combat influenza virus infection and cellular parameters (e.g., IFN-γ and IL-10 production, Granzyme B activity) might improve predicting clinical protection (49, 50). Therefore, the question remains whether the evaluation methods of influenza vaccine efficacy are currently optimal and truly reflecting the potential of vaccines in development as well as current vaccines. Evaluation of antibody specificity could benefit from further testing for functionality such as neutralization and ADCC (antibody-dependent cytotoxicity) (51).

### Adapting the Antigen Dose of Influenza Vaccines

To overcome low vaccine responses to influenza the vaccine antigen dose has been increased four times from 15 μg to 60 μg hemagglutinin (HA) per strain in a trivalent vaccine formulation for intramuscular use (Fluzone^®^). The higher antigen dose implicates a higher availability of the antigen, increase in antigen uptake by dendritic cells, antigen presentation to lymphocytes and consequently their activation resulting in a strengthened vaccine response. High-dose influenza vaccine lead to higher hemagglutinin antibody titers (HAI) and seroprotection rates in individuals over 65 years compared to standard dose vaccine (52). Otherwise, with regard to cellular immune responses PBMC of aged high-dose vaccine recipients produced significantly higher IL-10 levels after live influenza virus stimulation, which might question whether clinical protection is better than after standard dose due to the immunosuppressive properties of IL-10 (50). Results from two meta-analyses and systematic reviews show better protection with the high dose vaccine in the elderly based on a lower risk [relative risk (RR) 0.76] to develop laboratory-confirmed influenza when receiving the high-dose vaccine compared to the standard dose vaccine (53) and a higher relative vaccine efficacy against ILI (rVE 19.5%), hospitalization for influenza (rVE 17.8%), pneumonia (rVE 24.3%) as well as all-causes (rVE 9.1%) in high-dose vaccinated participants (54).

Another aspect of this high-dose vaccine is that it consists of a split virus that has been reported to induce better T cell immunity measured by IFN-γ production and by cytotoxicity assays *in vitro* than subunit vaccines (55) and to exhibit 33.5% higher protection against laboratory confirmed influenza in people aged above 50 seeking medical attendance for respiratory illness (56). Split virus vaccines lack some of the purification steps of subunit vaccines and therefore may contain a larger amount of internal proteins (55) that are important to elicit cellular CD8^+^ lymphocyte responses necessary for viral clearance (57).

Another high-dose influenza vaccine for intramuscular administration exists, containing three times the dose of the standard influenza vaccine. This vaccine (Flublock^®^) is recombinantly produced with the advantage of comprising 4 vaccine strains and therefore both influenza B linages. It is approved in the United States from the age of 18 years, however, not explicitly licensed or recommended for the older population due to insufficient efficacy data for those above 65 years of age. In a head-to-head comparison with a standard dose quadrivalent vaccine, though, geometric mean titers were non-inferior except for the influenza B Victoria linage in persons above the age of 50 (58).

Both high-dose vaccines as well as the MF59^®^ adjuvanted vaccines (see 2.3) increased GMT levels, seroprotection rates as well as IFN-γ^+^ CD4^+^, and CD8^+^ T cells for most time points studied compared to a non-adjuvanted standard dose vaccine in older adults (59).

### Changing the Route of Influenza Vaccine Application From Intramuscular to Intradermal

Since the dermis is rich in antigen-presenting cells (APC) such as dendritic cells (DC) and Langerhans cells (LC) intradermal (i.d.) application of vaccines is performed in order to facilitate antigen uptake by these APC and therefore also downstream adaptive immune responses following antigen processing and presentation (60). Along these lines i.d. vaccination can be utilized as a dose-sparing method, but it has been demonstrated that applying the same dose (i.e., 15 μg/strain) as the standard i.m. vaccines significantly improves GMT ratio compared to reduced doses (61). Intradermal influenza vaccination enhanced immunogenicity such as antibody responses and seroprotection rates compared to standard split or subunit vaccines in persons older than 65 (62). Yet, meta-analyses of RCT where reference groups received i.m. vaccination reported that i.d. application induced comparable seroconversion and seroprotection rates in older adults (63, 64). However, to which extent immunogenicity of i.d. administered vaccines might be affected by age-induced changes in the dermal structure and cellular composition needs further clarification. Additionally, how immunogenicity translates into clinical effectiveness has not been studied systemically so far and i.d. vaccine efficacy has been extrapolated from immunogenicity data (65). With regard to adverse events following immunization, higher reactogenicity at the injection site such as erythema and swelling has been described in comparison to i.m. administered vaccines, nevertheless they were classified as mild and transient with no differences in pain level (61, 64–66). Intradermal influenza vaccines have been used for several years, but are currently not available in many countries.

### Adjuvants for Influenza Vaccines

According to a systematic review and meta-analysis, non-adjuvanted inactivated influenza vaccines (split, subunit, and virosomal vaccine) show similar immunogenicity as expressed by geometric mean titers irrespective of age and disease status (67).

These results are surprising since the virosomal vaccine is considered not only as a delivery system but also as a vaccine adjuvant itself (68). Virosomes are able to mimic natural infection since receptor binding and membrane fusion is functioning and able to induce cellular and humoral immune responses but without the risk of virus replication since the viral genome is lacking (69). The virosomal vaccine showed increased immunogenicity compared to standard trivalent influenza vaccines (70). However, clinical efficacy and effectiveness data are lacking for the older population. The virosomal influenza vaccine is currently not being produced and therefore will not be discussed further in this review.

MF59^®^, a squalene-based oil-in water emulsion, was developed as an adjuvant for the seasonal influenza vaccine targeting persons above 65 (71). Its mode of action is linked to local proinflammatory cytokine production mediating cell recruitment, stimulating antigen uptake and cell differentiation of DCs as well as improving B cell differentiation and their persistence in germinal centers (72). Addition of MF59^®^ to a subunit influenza vaccine can enhance antibody production compared to standard vaccines even against drifted strains such as H3N2, that is linked to severe disease in the elderly (73–75). Additionally, use of MF59^®^ adjuvanted influenza vaccines can lead to expansion of predominantly IL-2 producing CD4^+^ lymphocytes against pandemic H5N1 compared to a non-adjuvanted vaccine (76). This effect was, however, not seen against seasonal influenza A/H3N2 in another season where rather Granzym B^+^ and Perforin^+^ CD4^+^ and CD8^+^ lymphocytes increased (57). According to a meta-analysis, the MF59^®^ adjuvanted vaccine is better in preventing pneumonia-associated hospitalizations [51% pooled adjusted vaccine efficacy (aVE)] and laboratory confirmed influenza (60.1% pooled aVE) than non-adjuvanted influenza vaccines in the elderly (71). Data form the 2018/19 influenza season in the United Kingdom, the first season after introduction of the adjuvanted influenza vaccines for persons above 65 years, demonstrated increased vaccine efficacy estimates in a test-negative study design in older adults vaccinated with the adjuvanted vaccine. Two different end-of-the-season evaluations were published showing a higher aVE against laboratory confirmed influenza with the adjuvanted vaccine (62.0%) compared to the overall aVE (49.9%) (77) and a aVE against influenza-related hospitalization of 53.8%. after receiving the adjuvanted vaccine, that, however, cannot be compared to those receiving standard influenza vaccines due to the very low number of participants in this group (78). Interestingly, in the latter analysis those vaccinated also in the previous 2017/18 season seemed to benefit from a higher aVE against hospitalization of 57% compared to 44.8% in those only vaccinated in the 2018/19 season. These results have to be assessed by taking into account the high influenza vaccine coverage of about 70% in the elderly as well as the universal influenza vaccine program for children between 2 and 9 years of age as part of the strategy to reduce infection also in the elderly risk group.

## Herpes Zoster: Combating a Latent Virus Infection (Live-Attenuated Versus Recombinant Adjuvanted Vaccine)

Primary infection with varicella-zoster virus (VZV) usually occurs in childhood and manifests as varicella (chickenpox). As a member of the herpes viruses, VZV establishes life-long latency in the sensory ganglia. Reactivation of VZV can occur throughout life, but is usually clinically asymptomatic as the reactivation is controlled by T cell-mediated immunity (CMI). When these immune responses decline below a critical threshold viral reactivation cannot be contained anymore. Retrograde viral spread through the sensory nerve to the innervated dermatome occurs, leading to herpes zoster (HZ). This results in the typical unilateral, segmented skin rash on the abdomen or the face, where affection of the eye can have severe consequences, and to dermatomal pain. The risk of HZ is elevated in patients with a compromised immune system due to hematological malignancies, HIV infection, chemotherapy or under immunosuppressive therapy (e.g., after transplantation or for autoimmune disease) (79). In addition, the risk of developing HZ increases substantially with age. The mean age at onset is 59.4 years with 68% of the cases occurring those 50 years and older. In the United States there are more than 1.1 million cases and in Europe about 1.7 million cases of HZ per year, respectively. The most common complication of HZ is post-herpetic neuralgia (PHN), which is defined as pain persisting or occurring more than 90 days after appearance of the rash. The incidence of PHN also increases with age. Among HZ patients older than 50 years the incidence of PHN is 18% and rises to 33% in HZ patients over 80 years (80). PHN can be prolonged (>6 months), and is often severe. Pain management is frequently difficult and of limited success and might be further complicated by co-medication and underlying diseases, which are common in the older population (81). In many cases PHN has a substantial impact on quality of live and activities of daily living (82), leading to a loss of independence and eventually to institutionalization. Prevention of HZ is therefore an important goal in order to improve quality of life for the older population and also helps to relieve health care and social systems.

The goal of vaccination against HZ is the restoration of the VZV-specific CMI, which was generated during primary infection. Therefore, the vaccine-induced immune response is mainly a boosting of memory responses rather than a primary response.

### Adapting Antigen Dose of the Live-Attenuated VZV Vaccine

The first vaccine against HZ was a live-attenuated vaccine, which contains the same VZV strain (Oka Merck strain) that is used as a childhood vaccination to prevent chickenpox. In comparison to the childhood vaccine, the adult vaccine (Zostavax^®^) contains 14-fold more viral particles. The vaccine is safe and has a modest and well-tolerated reactogenicity profile. Clinical efficacy of this vaccine was demonstrated in a Phase III randomized, double-blind trial enrolling 38,800 persons older than 60 years, which were followed-up for approximately 5 years. The vaccine was 51% efficient in preventing HZ and 67% in preventing PHN, respectively (83). Efficacy against HZ was age-dependent and dropped from 64% in the age group 60–69 years to 41% in the age group 70–79 years and <20% for persons older than 80 years. This age effect was confirmed in a separate study demonstrating an efficacy of 70% in a younger cohort (50–59 years) (84). Long-term follow-up showed that the protective effect of the vaccine waned over time and was lost approximately 10 years after vaccination (85). Immunological studies confirmed that vaccination increased VZV-specific immunity and that immunogenicity was negatively correlated with age. Vaccine-induced immune responses declined over the three years of follow-up. Both CMI and antibody levels correlated with the protective effect of the vaccine, but individually were not able to reliably predict protection. Therefore, neither parameter is suitable as a correlate of protection for further vaccine development (86). Re-vaccination after 10 years is feasible and results in a booster effect (87). The limited efficacy of this vaccine is somewhat surprising, as live-attenuated vaccines are usually eliciting robust humoral and cellular immune responses.

### Using a Recombinant Herpes Zoster Antigen With an Adjuvant

Recently, a novel recombinant vaccine against HZ (Shingrix^®^), which contains the viral glycoprotein E (gE) and the adjuvant system AS01B has been introduced in the United States, Canada, Europe, Japan, and elsewhere. gE is the major component of the viral envelope and gE-specific CD4^+^ T cells and antibodies are induced during natural infection. The adjuvant AS01B consists of 3-O-desacyl-4′-monophosphoryl lipid A (MPL), which is a detoxified derivative of *Salmonella minnesota* lipopolysaccharide and QS-21, a saponin found in the bark of the tree *Quillaja Saponaria* Molina, fraction 21. The two adjuvant components are formulated in liposomes, which are nanospheres of phospholipid bilayers acting as antigen delivery systems. MPL stimulates antigen-presenting cells via the toll-like receptor (TLR) 4 pathway and induces the expression of co-stimulatory molecules and the production of cytokines. QS-21 enhances antibody responses and promotes T cell responses in animal models (88). The molecular mechanisms underlying its adjuvant effect have only recently been partially deciphered (89). QS-21 targets subcapsular macrophages in the draining lymph node and activates caspase-1 (90), potentially independently of the NLRP3-inflammasome. As a liposomal formulation QS-21 enters antigen-presenting cells by cholesterol-dependent endocytosis followed by lysosomal destabilization and activation of Syk kinase (91). It has also been proposed that this process facilitates the escape of the antigen into the cytosol, where it can enter the MHC-I pathway (92). The AS01B-mediated activation of the innate immune system at the site of injection and in the draining lymph node is rapid and transient leading to efficient activation of adaptive immune responses (93). AS01B induces an INF-γ biased CD4^+^ T cell response with only moderate IL-5 production, high levels of T cell proliferation and IL-2 production (94). For optimal adjuvanticity all three components (MPL, QS-21, liposomes) are required together and in specific amounts as elucidated in comparative studies of different combinations in mice, and QS21 and MPL seem to work synergistic in some aspects (95). AS01B induces IFN-γ related pathways, which are not stimulated by either component alone (96).

Two randomized placebo-controlled Phase III clinical trials enrolling in total more than 30,000 participants older than 50 years or 70 years, respectively, were conducted in order to demonstrate efficacy of the vaccine (97, 98). Administration of two doses, 8 weeks apart resulted in 97.2% (95% CI: 93.7–99.0) protection against herpes zoster in persons over 50 years of age and did not significantly decrease in older age groups. These results were confirmed in the second trial, which included even older participants and allowed sub-group analysis for persons older than 80. Efficacy dropped slightly over time, but remained above 85% for the first 4 years after vaccination. Long-term follow-up is ongoing to determine the duration of protection. Efficacy against PHN was difficult to assess, as only very few cases of HZ occurred in the vaccinated group, but attenuation of pain was observed in these rare cases. Immunogenicity of the vaccine was analyzed in a sub-cohort of the large trials and following a peak response 4 weeks after the second dose, robust antibody and CD4^+^ T cell responses were found for at least 3 years after the vaccination. 75% of the cohort had antibody levels above the humoral response threshold (≥fourfold increase above baseline) after 3 years. The percentage of vaccinees with CD4^+^ T cells responses (≥fourfold increase above baseline) dropped from 93.3% (peak response) to 57.2% after 12 months and was then stable until month 36. Responding CD4^+^ T cells were defined by expression of at least 2 of the tested effector molecules (CD40 ligand, INF-γ, interleukin 2 and TNF). A slight negative effect of age on T cell responses was observed (99). The kinetics of gE-specific T cell responses were comparable to VZV-specific responses induced by the live-attenuated vaccine, but overall the immune response was stronger after the recombinant vaccine (86). Previous studies have also demonstrated a slight decrease of T cell, but not antibody responses with age (100–102) and gE-specific CD4^+^ T cell responses substantially above pre-vaccination levels for at least 9 years (103). In summary, the adjuvanted recombinant HZ vaccine seems to overcome the deleterious effects of immunosenescence and shows that a single antigen from a complex pathogen is sufficient to induce high levels of protection (104). The majority of adverse effects were transient reactions at the site of injection and systemic symptoms, such as headache, fatigue or myalgia were also relatively frequent. However, no major safety concerns were identified and after administration of more than three million doses within the first year after licensure there was no evidence of an increased risk of autoimmune reactions in response to the adjuvant system, which was raised as a potential concern (104).

An additional advantage of the recombinant vaccine is its suitability for immunocompromised patients. As mentioned above, these individuals are at high risk to develop HZ and the live-attenuated vaccine cannot be used for them. The adjuvanted recombinant vaccine has been evaluated in patients suffering from different types of immunocompromising conditions. Safety and immunogenicity have been demonstrated in patients after renal transplantation (105) and in HIV-positive patients. However, the number of subjects with low CD4 counts were too low to formally demonstrate immunogenicity in this subgroup of HIV-positive patients (106). Clinical efficacy of the recombinant vaccine was 68.2% (95% CI 55.6% to 77.5%) in adult patients after autologous hematopoietic stem cell transplantation (107). In addition the maximum worst pain score and the activities of daily living scores were improved in the patients who developed HZ despite vaccination compared to HZ cases in the placebo group indicating that the severity of break-through HZ cases is reduced (108). In patients receiving two doses of the recombinant vaccine at the start or after completion of chemotherapy for hematologic malignancies vaccine efficacy against HZ was 87% in the first year after vaccination (109). Vaccination before chemotherapy against solid tumors elicits higher immune responses compared to vaccine administration at the start of therapy (110). Safety profiles were acceptable in all of these studies. Overall these results suggest that the recombinant adjuvanted vaccine against HZ can be safely and successfully used in various patient groups under immunosuppression.

## *Streptococcus Pneumoniae*: Targeting Different Serotypes of a Colonizing Bacterium (Polysaccharide Versus Conjugate Vaccine)

There are more than 90 distinct serotypes of *Streptococcus pneumoniae* (pneumococcus), classified based on their polysaccharide capsule, which also serves as an essential virulence factor. Only a limited number of serotypes of these gram-positive diplococci are pathogenic (111). Disease manifestations can be non-invasive (otitis media, sinusitis, conjunctivitis, pneumonia) or invasive (bacteremic pneumonia, meningitis, sepsis). The World Health Organization has classified *S. pneumoniae* among the top 12 bacterial pathogens for which research and development of new antimicrobial strategies should be promoted (112). Incidence rates of community acquired pneumonia (CAP) rises dramatically with age with estimated rates ranging from 18.2 per 1000 person-years in people aged 65–69 years, to as high as 52.3 per 1000 person-years in those aged over 85 years. *S. pneumoniae* is the most frequently isolated pathogen in this age group. Among United States adults aged 50 years or older, nearly 30 000 cases of invasive pneumococcal diseases (IPD) and over 500 000 cases of non-bacteremic pneumococcal pneumonia were estimated to occur yearly, resulting in more than 25 000 pneumococcus-related deaths (113). Antimicrobial resistance of *S. pneumoniae* is an increasing problem (114). Development of pneumococcal penicillin resistance several decades ago shifted antibiotic use in suspected cases toward macrolides. This strong selective pressure contributed to the spread of macrolide-resistant *S. pneumoniae.* (115). In addition, 20–40% of isolates are resistant to clindamycin or trimethoprim-sulfamethoxazole. Resistance to fluoroquinolones is lower, but similar to resistance to tetracyclines has increased (116). The dissemination of multi drug resistant (MDR) clones is of particular concern. A very limited number of MDR clones cause the majority of antibiotic resistant *S. pneumoniae* infections worldwide (117). In order to counteract this alarming development, strategies to prevent infection, instead of treating cases are crucial.

Asymptomatic carriage, which means a transient colonization of the upper respiratory tract by *S. pneumoniae* is frequent in children (up to 85%), but less prevalent in the elderly (approx. 20%). Study results vary greatly, due to differences in detection methods and sampling sites (118). In the nasopharynx *S. pneumoniae* interferes with host responses, such as the complement system, the recruitment of neutrophils and the protective mucus layer and competes/interacts with the local microbiome (119). In the elderly pneumococcal pneumonia has been linked with a disturbed respiratory tract microbiome, but it is unclear whether this is the cause or consequence of pneumococcal colonization (120). Pneumococcal disease occurs when the colonizing bacteria reach tissues of the lower respiratory tract, the ear or the eye or enter the blood stream. The pneumococcal toxin pneumolysin, but also Influenza A virus can damage the respiratory epithelia leading to bacterial spread and pathologies (121, 122). Bacterial co- or secondary infection is a frequent complication of influenza infection. The exact numbers of co-infections vary greatly in different studies, but range between 11 and 35% in most cohorts. *S. pneumoniae* was the most common pathogen, which accounted for 35% (95% CI: 14–56%) of the bacterial co-infections analyzed in this meta-analysis (123).

Polysaccharide-specific antibodies can be measured by ELISA, which measures the amount of antibodies binding to the antigen. This assay has some limitations, as it measures only IgG, but not other antibody classes. Many individuals have high levels of naturally acquired antibodies, which makes it difficult to define a correlate of protection. First generation ELISA methods were frequently unspecific, but this issue has been solved by improving the protocols (124). In addition, many studies measure functional antibodies by opsonophagocytosis assay (OPA). In young children strong correlations between ELISA and OPA titers have been observed, but in the elderly and in immunocompromised patients correlations are poor (125). Considerable waning of opsonizing antibodies has been observed 6 years after vaccination in a cohort of frail elderly, despite persistence of IgG antibodies detectable in ELISA (126). It has been demonstrated that older adults have a lower capacity to opsonize pneumococcal bacteria, despite sufficient IgG concentrations, as measured by ELISA. This is probably due to a lack of IgM antibodies with opsonizing function as IgM-producing memory B cells decline with age (127). Naturally acquired humoral immune responses also decline with age, particularly when measured by OPA (118). In some cases, cross-reactivity of antibodies with other serotypes can be seen in ELISA-assays (e.g., anti-19F antibodies elicited by PCV-7 binding serotype 19A polysaccharides), but not in OPA. Vaccination with 19F does not provide clinical cross-protection against 19A highlighting the relevance of the OPA results (128). In addition, OPA titers seem to better predict vaccine failures than ELISA measurements (129). Therefore, in various settings OPA-measurements are crucial and probably provide a more robust correlate of protection, despite that fact that they are more complex, more expensive and less standardized. The development of multiplex technologies made OPA more suitable for larger studies and analysis of a larger number of serotypes (130). While an opsonic titer of 1:8 is used as a threshold to define immunogenicity of pneumococcal vaccines in children, 1:64 was suggested for adults (131). This threshold is still controversial and most studies rely on determining “relative” immunogenicity, by comparing two experimental groups e.g., different vaccines or age groups. Local antibody responses in the respiratory tract might be of great interest in the context of pneumococcal vaccination. Induction of anti-polysaccharide antibodies in saliva and tears after pneumococcal vaccination has been described. Interestingly, the increase in IgG and IgM was more pronounced than the IgA response. Nasal secretions were not analyzed in this study and the functional and clinical effects of local antibodies are unclear (132). It has been postulated that IgA antibodies are of limited importance for protection, as all pneumococci synthesize an IgA1 protease, abrogating the protective effect of this antibody class (133).

The first vaccines against *S. pneumoniae* were polysaccharide vaccines (PPV) containing the purified bacterial capsule polysaccharides. The currently available 23-valent polysaccharide vaccine (PPV-23) was licensed for adults in the early 1980s. It contains the serotypes 1, 2, 3, 4, 5, 6B, 7F, 8 9N, 9V, 10A, 11A, 12F, 14, 15B, 17F, 18C, 19A, 19F, 20, 22F, 23F, and 33F. Purified polysaccharides are T cell independent antigens, and as such elicit a distinct immune response. Direct cross-linking of the B cell receptor activates B cells and drives differentiation into plasma cells, which produce antibodies. Due to the lack of T cell help this process happens independently of germinal centers and results in short term antibody responses, which are mainly IgM and IgG_2_. Memory B cells are not generated in the course of these immune responses (134) and the B cell pool is depleted of the relevant specificities potentially leading to hyporesponsiveness to subsequent vaccine doses (135). Based on studies in mice it has been hypothesized that marginal zone B cells play a crucial role in this type of immune responses (136). As mentioned above, burden of pneumococcal disease is highest in the elderly and in infants. As the immune system of infants is not able to elicit immune responses to most polysaccharide antigens in the first 2 years of life (137, 138), the PPV-23 vaccine is not suitable for young children and has only been licensed for adults. PPV-23 has been used in older adults since its licensure and many countries specifically recommend this vaccine for the older population.

### Conjugation of Polysaccharide Antigens

Chemical conjugation of polysaccharides to carrier proteins enables uptake and processing of the protein component by polysaccharide-specific B cells. Carrier-specific peptides are then presented in the context of MHC II molecules, which can be recognized by CD4^+^ T cells. Thereby, carrier-specific T cells can provide T cell help to polysaccharide-specific B cells eliciting T cell dependent immune responses to polysaccharide antigens (135). As a result, memory B cells are generated enabling an anamnestic response upon booster vaccination. Class switch and avidity maturation can take place, and conjugate vaccines are immunogenic in infants. The first conjugated pneumococcal vaccine contained 7 serotypes (PCV-7; contains serotypes 4, 6B, 9V, 14, 18C, 19F, and 23F), used Crm-197, a derivative of diphtheria toxoid as a carrier protein, and was introduced in the late 1990s/early 2000s for young children. As a consequence, incidence of invasive pneumococcal disease (IPD) caused by the serotypes present in the vaccine dropped substantially in the targeted age group. Interestingly, incidence of IPD also declined to a lesser degree in the older population due to herd immunity effects. PCV does not only prevent disease in children, but also precludes carriage of *S. pneumoniae*, potentially stopping transmission from children to older adults. However, a slight increase of cases was observed for serotypes not included in the vaccine (serotype replacement) for children as well as for older adults. Of particular concern was the substantial increase of cases caused by serotype 19A (139). The next generation of conjugated pneumococcal vaccines contained 10 (PCV-10; PCV-7 serotypes plus 1, 5, and 7F, conjugated to non-typeable *Haemophilus influenzae* protein D, diphtheria or tetanus toxoid) or 13 (PCV-13; PCV-10 serotypes plus 3, 6A, 19A, conjugated to Crm-197) serotypes. Similar to PCV-7 introduction of these vaccines into routine childhood vaccination programs reduced the incidence of IPD caused by the (now more) serotypes covered by the vaccines in children and older adults. But serotype replacement was again observed not only in the pediatric setting, but also for older adults. As an example, the incidence of IPD per 100.000 persons (≥65y) in England and Wales was 24.67, 14.97, and 6.25 for PCV-13 serotypes in 2000–2006, 2008–2010, and 2016–2017, respectively, while at the same time the incidence of non-PCV-13 serotypes increased from 9.55 to 12.76 and 22.68 (140). The exact epidemiological situation is very heterogenous in different countries due to regional differences in childhood vaccination programs, vaccination coverage, transmission dynamics etc., and serotype replacement was less pronounced in other countries.

Immunogenicity of PPV-23 declines with age. After vaccination with PPV-23 antibody responses of older adults showed alterations in class and subclass usage as well as differences in somatic hypermutation compared to young individuals (141). In addition, the opsonophagocytic activity was lower in older adults (131, 142).

In contrast to PCV-7 and PCV-10, which are only licensed for children, PCV-13 is approved for all age groups (children, young and old adults). However, it has to be pointed out, that conjugate vaccines were primarily developed for use in children and not in older adults. First comparisons of immunogenicity between polysaccharide and conjugated vaccines in older adults have already been performed using PCV-7. One study demonstrated that PCV-7 induces higher antibody levels (ELISA and OPA) in persons older than 70 years, receiving a pneumococcal vaccine for the first time (143). Other studies described similar antibody responses for both vaccines (144–146). It has to be taken into account that the patient populations in these studies were very heterogenous, that for most studies sample size was relatively small and that previous vaccination with PPV-23 has an impact on immunogenicity. One study using only PPV-23 showed that antibody levels measured by ELISA at the time of enrollment were higher in persons who had received PPV-23 more than 3 years prior to the study. Upon vaccination their antibody response was slightly lower than that of the cohort vaccinated for the first time (147). Increasing the dose of PCV-7 by twofold resulted in significantly higher OPA titers for five of the seven serotypes compared to PPV-23 (148), highlighting the potential benefit of higher antigen dose, as also shown for influenza (see section “Adapting the Antigen Dose of Influenza Vaccines”). Frailty has been associated with lower antibody responses to pneumococcal vaccine (144). More recent studies compare PPV-23 and PCV-13, and systematic meta-analyses (149, 150) showed significantly higher antibody levels for 10 of the 13 serotypes after vaccination with PCV-13. It has to be mentioned, that 6A is not contained in the 23-valent, but only in the 13-valent vaccine and therefore obviously the 13-valent vaccine is superior for this serotype. For the remaining 3 serotypes (3, 7F, and 14), both vaccines are equally immunogenic.

Of particular interest is also the potential benefit of sequential vaccination with both vaccines and/or of repeated vaccination in regular intervals. Early studies using PCV-7 showed that a second dose of PCV-7 1 year after either PCV-7 or PPV-23 leads to similar or slightly higher responses compared to the first vaccination (143). Sequential vaccination was beneficial for long-term maintenance of antibodies, as OPA titers waned already within the first year in persons receiving only PPV-23, but not in a cohort who received PCV-7 followed by PPV-23 (145). In a meta-analysis on PCV-13, prior vaccination with PPV-23 did not influence the immunological response to the conjugate vaccine (149). Safety profiles were comparable for PCV-13 and PPV-23 in this analysis.

However, the clinically relevant parameters are of course efficacy and effectiveness. These parameters have been studied mainly for invasive pneumococcal disease (IPD), which includes bacteremic pneumonia, sepsis and meningitis and in some studies also for pneumococcal pneumonia. A systematic meta-analysis showed pooled vaccine efficacy/effectiveness (VE) of PPV-23 against IPD of 73% (95% CI: 10–92%) in clinical trials, 45% (95% CI: 15–65%) in cohort studies and 59% (95%CI: 35–74%) in case-control studies (151). In the same analysis VE against pneumococcal pneumonia was 64% (95% CI: 35–80%) and 48% (95%CI: 25–63%) in clinical trials, or cohort studies, respectively. This is in contrast to other meta-analyses, which did not demonstrate efficacy against pneumonia (152–155). The discrepancy could be explained by the fact that Falkenhorst et al. excluded several studies in their analysis, which had a high risk of bias, because of insufficient specificity of the antibody test used to diagnose cases of pneumococcal pneumonia. Higher VE estimates in clinical trials compared to observational studies might be due to the differences in follow-up times, which were shorter for the clinical trials, suggesting waning protection over time. Clinical efficacy of PCV-13 was demonstrated in a large Phase IV randomized, placebo-controlled trial involving more than 84,000 older adults (156). In the per-protocol analysis VE against first episodes of community-acquired pneumonia (CAP) caused by vaccine-type strains was 45.6% (95% CI: 21.8–62.5%) and against vaccine-type IPD 75.0 (95% CI: 41.1–90.8%), respectively. The protective effect was consistent for the 4-year follow-up period. No reliable data is available regarding the clinical efficacy of vaccination strategies combining both types of vaccines or of repeated doses of either vaccine.

It is still debated which pneumococcal vaccination strategies provides optimal protection for the older population. PPV-23 covers more serotypes, but does not induce long-lasting and memory responses and might induce tolerance or hyporesponsiveness upon repeated vaccination, similar to the meningococcal polysaccharide vaccine (157). PCV-13 induces stronger antibody responses, which seem to be longer lasting and might offer boosterability. However, due to routine childhood vaccination with PCV13 and the associated herd immunity effects the incidence of pneumococcal disease caused by the PCV-13 vaccine serotypes decreases in the older population, while other serotypes prevail (serotype replacement). This controversy is also reflected in heterogenous vaccination recommendations, as various countries e.g., in Europe recommend either PPV-23, or PCV-13 or a combination of both, which tries to exploit the advantages of both vaccines. Vaccination recommendations in the United States have very recently been adapted. After recommending sequential use of both vaccines for several years, now only PPV23 is generally recommended and the addition of PCV should be considered for the individual patient in a shared decision process (158). These uncertainties might contribute to the still low vaccination coverage in many countries (159–162). Development of conjugate vaccines comprising more serotypes is ongoing and it might be worthwhile to consider the option of including different serotypes in pediatric compared to adult vaccines in order to reflect the distinct pattern of serotype prevalence in the different age groups.

## Future Perspectives to Improve Vaccination of the Older Population

### Universal Vaccines

Pathogens that undergo constant antigenic changes (e.g., influenza) as well as those encompassing a large antigenic diversity (e.g., pneumococcus serotypes) call for vaccines, which target all antigenic variants in order to be broadly protective. Clinical data emphasize the need for improved influenza vaccines. The diverse health status of the aged population due to underlying diseases including past influenza history, level of immunosenescence and medication makes it difficult to find a “one shot that fits all” solution. The high variability of the pathogen itself further aggravates the difficulties of developing an optimal vaccine. In order to tackle the antigenic variability, the development of a universal influenza vaccine is appealing. The aim of the universal influenza vaccine is to induce broadly neutralizing antibodies to highly conserved epitopes, FcR mediated effector functions or cellular cytotoxicity mechanisms and therefore longer-lasting immunity over several seasons ideally against influenza A and B (42, 163). Different approaches are currently tested in clinical trials such as chimeric HA proteins in prime-boost trials, stem-based immunogens, virus-like particles, peptides against conserved epitopes and nucleic acid platforms (51, 164). Clinical evaluation will reveal whether neutralizing antibodies will be induced, which are able to prevent influenza infection. Results from murine studies with regard to stem-based immunogens are promising, but await further testing in humans (165, 166). A different approach is to boost T cell-mediated cytotoxicity to eliminate virus-infected cells thereby mitigating infection and ideally keeping it asymptomatic (167). It is furthermore yet unclear whether universal influenza vaccines will need to be enhanced by adjuvants or adaptation of the antigen dose to induce sufficient immunity in the older population.

Serotype replacement has been observed after introduction of conjugated pneumococcal vaccines (see section “Conjugation of Polysaccharide Antigens”) and next-generation conjugated vaccines containing additional serotypes are being developed. Immunogenicity and safety of a 15-valent conjugated vaccine has been shown to be comparable to PCV-13 in early stage clinical trials (168). However, universal vaccines against *S. pneumoniae* would hopefully be able to fully overcome the risk of serotype replacement and would therefore probably have a more profound long-term clinical impact. An engineered pneumococcal strain, which lacks a polysaccharide capsule and toxins has been tested as an inactivated whole cell vaccine in mice (169, 170). In combination with alum and administered subcutaneously to mice this whole cell pneumococcal vaccine induces antibodies binding to different encapsulated strains, activating the complement system and inducing phagocytosis of the bacteria *in vitro* (171). In a Phase I clinical trial in healthy adults this vaccine elicited antibodies to a range of pneumococcal proteins including multiple conserved antigens, as well as T cell responses (172, 173). Recently, a multiple-antigen pneumococcal vaccine, which utilizes TIGR4 whole cell lysates enriched for surface proteins by chromatography was shown to induce robust antibody response against several serotypes and protected mice against pneumonia (174).

An alternative approach for a universal pneumococcal vaccine is the use of individual proteins. Anti-protein immune responses have been described following colonization (175), but are reduced in old age (176). The majority of candidate proteins are virulence factors and well-conserved surface proteins. Various potential vaccine proteins are investigated in pre-clinical studies and several have already been tested for safety and immunogenicity in humans. Most of these candidate vaccines use pneumococcal histidine triad protein D (PhtD), detoxified pneumolysin derivative (PlyD) and pneumococcal surface protein (PspA) alone or in different combinations and are immunogenic in humans while showing acceptable safety profiles (177, 178). As a next step highly conserved protein fragments or peptides were investigated as potential vaccine antigens and showed immunogenicity and protective effects in mouse models (179). Further clinical studies are needed to demonstrate immunogenicity and ultimately clinical efficacy in humans.

### Induction of Secretory IgA Antibodies and Mucosal Delivery

Another approach to improve immunity to pathogens that enter via the mucosal surfaces such as influenza is to stimulate the production of secretory IgA (sIgA) in the upper respiratory tract. sIgA is able to neutralize the virus at the entry site and better cross-protective properties to variant strains have been described (180). Although data suggest that i.m. and s.c. vaccination is able to induce limited amounts of mucosal IgA mucosal (intranasal) administration of the influenza vaccine can improve this effect (181). An intranasally administered virosomal influenza vaccine adjuvanted with LT (heat labile enterotoxin of *Escherichia coli*) has been withdrawn during the 2000/2001 season shortly after marketing due to safety issues as Bell palsy cases increased 19-fold (due to accumulation of LT in the olfactory bulb) (182, 183). Two other intranasally applied formulations have been tested, a live attenuated influenza vaccine (LAIV), which is also commercially available in many countries and a whole virus inactivated vaccine (WIIV). The LAIV vaccine is, however, not licensed for adults above 49 years of age due to safety concerns. Additionally, immunogenicity against the included H1N1 pandemic strain dropped during the 2013/14 season and therefore LAIV was intermittently not recommended by several national guidelines. The safety issues could be overcome by using the WIIV, which has intrinsic TLR7 signaling capacity (184). This vaccine is currently under clinical investigation and has been shown to induce serum IgG as well as sIgA responses without the need of a mucosal adjuvant in vaccinees younger than 60 years (185). Recently, it has been shown in the murine system that i.n. administered WIIV not only induced IgA but also boosted non-neutralizing antibodies and IFN-γ^+^ CD4^+^ T cells with cross-protective properties, especially if provided with an adjuvant (186). How this vaccine performs in older adults has not been tested yet. These results will be interesting since in aged individuals the response to influenza in respiratory epithelial cells is lower with respect to antigen processing and presentation (187).

Many pneumococcal vaccine candidates are investigated for mucosal delivery using intranasal, pulmonary, sublingual and oral administration routes. In the experimental human pneumococcal carriage (EHPC) model, live pneumococci are delivered intranasally to volunteers. This leads to the generation of antibody and Th17 T cell responses, independent of the occurrence of colonization and suggests that mucosal delivery of pneumococcal antigens is a promising strategy for vaccination (188, 189). The first generation of mucosal pneumococcal vaccine candidates used intranasal immunization with pneumococcal proteins or non-protein antigens such as phosphorylcholine, cell wall polysaccharide or capsular polysaccharides in combination with adjuvants based on bacterial toxins. Due to safety concerns, non-toxin based vaccine delivery systems are currently favored. Alveolar and bronchial administration of the licensed 23-valent polysaccharide vaccine using different inhaling devices was tested in healthy volunteers and in patients with chronic obstructive pulmonary disease. The administration was safe, but antibody levels were lower than after intramuscular injection with one device and not detectable in another study (190–192). In addition, live recombinant bacteria (see section “Activation of Cell-Mediated Immune Responses”), outer membrane vesicles from recombinant *Salmonella* and bacteria-like particles derived from *Lactobacillus lactis* have successfully been tested as pneumococcal vaccines in mice. An attenuated *Salmonella Typhi* strain expressing PspA, however, failed to induce anti-PspA antibodies after oral administration in an early stage clinical trial (193), highlighting the difficulties to translate results from animal models to humans. Novel approaches for mucosal delivery of pneumococcal vaccines include nanoparticles, which facilitate antigen uptake and release and trigger innate immune responses, as well as nanogels, which further prolong exposure to the antigen. No data on immunogenicity in humans is available so far. A comprehensive review of mucosal pneumococcal vaccine candidates has recently been published (194).

### Search for Novel Adjuvants

The requirements for novel adjuvants are to counteract the lower responsiveness of the innate and adaptive immune system to vaccines and the faster decline of protection due to immunosenescence as well as to counterbalance the low-grade inflammatory state that might hamper vaccine responses (195). MF59^®^ and AS01B are currently the only adjuvants explicitly licensed for persons older than 65 years (see section “Adjuvants for Influenza Vaccines” and section “Using a Recombinant Herpes Zoster Antigen With an Adjuvant”). MF59^®^ has been used for many years in the trivalent influenza vaccine and is currently further evaluated in a tetravalent influenza formulation in phase III studies in order to target both circulating influenza B linages (NCT02587221 and NCT03314662). In addition, different new adjuvants are evaluated for this age group at present which were reviewed in depth recently (72). AS03, another oil-in-water based adjuvant that has been approved for the pandemic influenza vaccination in 2009 led to higher antibody titers and seroprotection levels in the elderly compared to whole virion vaccine (196). AS03 induces chemokine and cytokine production thereby increasing the influx of inflammatory cells locally and to the draining lymph node (197). Furthermore, also specific CD4^+^ T helper cells with cross-reactive capacity and specific memory B cells were stimulated resulting in an overall longer antibody persistence than in recipients of non-adjuvanted vaccine (198). Montanide (ISA 51), an oil-in-water emulsion, has been applied with TIV in a phase I trial to adults between 55 to 75 years, however, results are not yet published (NCT01010737). Its mode of action is based on depot formation, stimulation of inflammatory signals and enhancing lymphocyte interaction in lymph nodes leading to increased antibody levels and vaccine antigen-specific CD8^+^ lymphocyte responses (199). Matrix M^TM^, a saponin based adjuvant, is currently tested for immunogenicity and safety in the elderly (NCT04120194 and NCT03293498) and enhances immune response by increasing leukocytes and their activation in lymph nodes and spleen in a murine model (200). Imiquimod, a TLR7/8 agonist able to strengthen innate danger signals and thereby activation of APC, applied as an ointment prior to intradermal trivalent influenza vaccination could elicit higher antibody titers, seroconversion and long-term seroprotection over a year in older adults with comorbidities (201). Another influenza vaccine adjuvanted with a TLR5 agonist reported high levels of antibodies and seroprotection in persons over 65 years but did not include a control group without the adjuvant (202). Several other adjuvants such as cytokines or T cell stimulating adjuvants might be good candidates to boost influenza-specific immune responses in older adults, but have not yet been tested in this age group or other high-risk groups. Still, if these adjuvants are added to seasonal vaccines, they have the shortcoming of inducing strain-specific immunity and therefore still need annual adaptation.

Several adjuvants and delivery systems have also been tested together with pneumococcal proteins. The adjuvant AS02V (oil-in-water emulsion combined with MPL and QS21) enhances humoral and cellular immune responses to the pneumococcal protein PhtD, PhtD-dPly and an 8-valent conjugated polysaccharide formulation in young and older adults (203, 204). DNA-based adjuvants, such as a plasmid encoding the Flt3 ligand and CpG oligonucleotides (TLR9 ligand) successfully enhanced mucosal immunity against a PspA-based protein vaccine in aged mice (205, 206). Other TLR agonists have also been shown to enhance immune responses and protection in mouse models (207, 208). Several other adjuvants and advanced delivery systems with the potential to increase efficacy of pneumococcal protein and peptide vaccines are in pre-clinical development (179).

The adjuvant 1018, containing a TLR9 agonist, has been recently approved by the FDA in the context of a hepatitis B vaccine. Primary vaccination of healthy individuals between 40 and 70 years of age led to higher seroprotection and antibody titers after a 2-dose regimen at 0 and 4 weeks compared to the licensed alum-adjuvanted vaccine containing the same antigen amount (20 μg HBsAg) applied three times at weeks 0, 4, and 24 (209). Although seroprotection rates were lower in the 60 to 70 year old participants, levels were still higher with the 1018 containing vaccine compared to alum (210).

### Activation of Cell-Mediated Immune Responses

Reinforcing the cellular immune responses after immunization may be worthwhile for specific pathogens. In case of influenza, CD8^+^ responses target highly conserved protein epitopes and are therefore naturally heterosubtypic (211). Therefore, utilizing reverse vaccinology approach to identify a peptides targeting and promoting cross-reactive effector and memory CD8^+^ responses could be promising to attain better protection even in already primed individuals. CD4^+^ lymphocytes also play an important role in influenza infection as they provide help to B and CD8^+^ cells as well as by mediating cytotoxic activity (212). Especially preexisting influenza-specific CD4^+^ T cells against conserved internal proteins could limit virus replication and alleviate symptoms (213). CD4^+^ cells also might undergo imprinting that possibly influences their lung homing capacity and therefore effector function upon influenza infection (214). Since T cell responses have non-sterilizing effects on influenza it will be important to generate influenza vaccines that promote both humoral and cellular responses to reach high levels of protection. This could be achieved by vector-based vaccines with adenovirus and modified vaccinia virus Ankara, which are already in clinical testing and might also be combined with seasonal influenza vaccines (212). Although these vaccines are attenuated or even replication deficient some are considered as live vaccines, which would limit their use for certain risk groups. In addition, preexisting immunity to the vector might hamper immune responses toward the target (215). Vector-based approaches using e.g., recombinant *Mycobacterium bovis* BCG strains, attenuated *Salmonella* strains or lactic acid bacteria have also been tested for pneumococcal vaccine (216, 217). In addition to protein-specific antibodies, CD4^+^ T cell responses might play an important role for protection against pneumococcal disease. It has been postulated that Th17 T cells are responsible for preventing colonization, whereas antibodies are more important in preventing invasive disease (216). The frequency of tonsillar regulatory T cells is lower and the number of Th17 T cells is higher in young adults compared to children. At the same time, the rate of pneumococcal carriage decreases with age (218). No data are available on older adults yet. In a mouse model, Th17 cells producing IL-17 are generated during pneumococcal infection and are responsible for subsequent protection against heterologous strains (219). Therefore, vaccines eliciting mucosal protein-specific Th17 responses might be a promising strategy toward a universal pneumococcal vaccine. Several vaccine candidates have been demonstrated to elicit Th17 biased T cell responses, which prevent pneumococcal colonization of mice (207, 220).

### Modification of the Vaccine Response by Senolytic and Immunomodulatory Drugs

A new strategy to overcome age-related changes in immune responses has emerged by studying small molecules that either lead to apoptosis of senescent immune cells or exhibit immunomodulatory effects (221, 222). Studies so far focused on chronic diseases affecting the older population and were mainly performed in animal models. It was speculated that these drugs might increase immune responses to vaccines and results will help to understand which cytokine networks and/or signal transduction pathways could be exploited to optimize vaccine responses in the older adults. First results in humans are promising as daily treatment with a combination of two mTOR inhibitors enhanced antibody titers against all three strains of a trivalent influenza vaccine by more that 20% in individuals aged above 65 years (223).

## Conclusion and Outlook

Not everybody responds to vaccines in the same way. The concept of personalized vaccinology -similar to personalized therapies in cancer patients- has been discussed extensively over the last years (224–226). Defining optimal vaccination (dose, route of administration, adjuvant etc.) for everybody individually, seems to be a promising strategy to ensure optimal protection with minimal side effects for everybody. However, there is still a long way to go to reach this goal. Systems biology and Omics-technologies have been employed to study individual vaccine-induced immune responses in detail and to identify common patterns (227, 228). Several studies were performed to identify predictive markers for vaccination success. Inflammatory responses and augmented B cell responses before vaccination were moderately accurate predictors of poor or stronger responses of older adults to Hepatitis B vaccination, respectively (229). Several T cell parameters including regulatory and PD-1 expressing T cells were identified as predictors for VZV-specific T cell responses induced by the live-attenuated VZV-vaccine (230). In middle-aged adults, naïve and regulatory T cells as well as low IL-1Ra levels were suggested as predictive markers for antibody responses after primary meningococcal vaccination (231).

**FIGURE 1:**
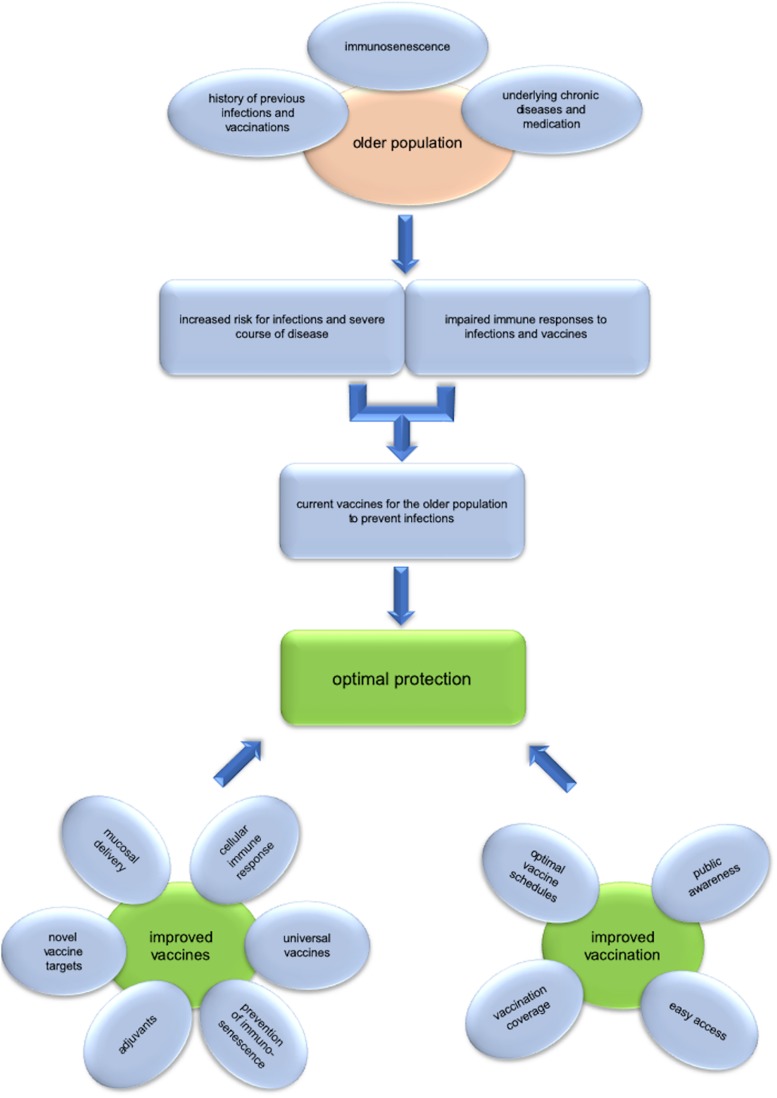
Challenges and opportunities of vaccination in old age. The older population is at increased risk for infections and severe course of disease, while immune responses to infections, and vaccines are impaired. Several factors, such as immunosenescence, underlying chronic diseases and medications as well as the history of previous infections and vaccinations influence the risk for the individual and lead to heterogeneity within this age group. Current vaccines for the older population are an efficient measure for preventing infectious diseases, but there is still much room for improvement. For optimal protection of the older population improved vaccines need to be developed. This includes the use of adjuvants, vaccines against additional pathogens, universal vaccines targeting variable pathogens, induction of cellular immune responses, mucosal delivery, and the prevention of immunosenescence. Of equal importance are strategies to improve vaccination. Optimal vaccine schedules and improved vaccination coverage rates are essential to maximize the benefit we draw from vaccination. Public awareness and knowledge as well as easy access to vaccination are crucial to reach these goals.

There is still a lot of room for improving vaccination for the older population (Figure 1). Novel vaccines are needed to target the many infectious diseases causing substantial morbidity in the older population, for which no vaccines are available so far. Respiratory syncytial virus (RSV) causes severe lower respiratory tract infections in vulnerable groups, such as infants and older adults. Several vaccine candidates have been shown to be immunogenic and safe, but failed to provide protection for older adults in clinical trials (232). Novel vaccine candidates might be developed based on recent discoveries regarding the structure of RSV proteins and the specific immune responses required for protection. Nosocomial infections are frequent in the older population and increasing rates of antibiotic resistance are a tremendous concern. Vaccines against these pathogens (e.g., *Clostridium difficile*, *Staphylococcus aureus etc.*) could have a substantial impact and are extensively studied (233). In addition, vaccines against many more pathogens, such as *Candida* spp., *E. coli* causing recurrent urinary tract infections etc. would be highly desirable and are under development (234, 235). Various approaches to improve the vaccines currently recommended and used for the older population have been discussed in detail above and many more are in early stages of development. A more detailed knowledge about age-related changes of the immune system will enable us to rationally design vaccines, which specifically target the aged immune system and are hopefully able to overcome its limitations. However, it needs to be pointed out that a first step toward improving protection of the older population is the optimal use of the currently available vaccines. Since antibody levels decrease faster in the elderly (236, 237, 240), some countries recommend shorter booster intervals for several routine vaccines (diphtheria, tetanus, pertussis, hepatitis B, tick-borne encephalitis) for older adults (238). However, primary vaccination schedules are usually not adapted for older adults, with the exception of a 3 + 1 scheme for tick-borne encephalitis vaccination in Sweden for those over 50 years (239). Additionally, more data are required on how primary and booster vaccinations perform in older patients with underlying chronic diseases that could result in lower vaccine responses and therefore protection. But even perfectly designed vaccines can only work, when administered to the population. Low vaccination coverage, although heterogeneous amongst countries, is still a major limitation. In order to provide immunity and protection of the individual and to establish herd immunity where applicable vaccination coverage rates need to be increased. Approaches to achieve this would include financial coverage and easy access. Additionally, awareness needs to be raised that vaccination is important for all age groups, which could be attained by education of medical personnel and decision makers as well as increasing health literacy in the general public. Finally, clear recommendations, ideally harmonized within Europe, would be helpful to guide those who administer vaccines and to improve acceptance.

## Author Contributions

AW and BW wrote and discussed the manuscript and agreed to be accountable for the content of the work.

## Conflict of Interest

The authors declare that the research was conducted in the absence of any commercial or financial relationships that could be construed as a potential conflict of interest.
